# Evaluation of Personality Features and Mental State of Keratoconus Patients

**DOI:** 10.14744/bej.2021.24482

**Published:** 2021-12-17

**Authors:** Mehmet Gokhan Aslan, Mert Besenek, Hasan Akgoz, Muhammed Fatih Satılmaz, Cicek Hocaoglu

**Affiliations:** 1. Department of Ophthalmology, Recep Tayyip Erdogan University Faculty of Medicine, Rize, Turkey; 2. Department of Child and Adolescent Psychiatry, Recep Tayyip Erdogan University Faculty of Medicine, Rize, Turkey; 3. Department of Psychiatry, Recep Tayyip Erdogan University Faculty of Medicine, Rize, Turkey

**Keywords:** Corneal topography, depression, keratoconus, obsessive-compulsive disorder, personality

## Abstract

**Objectives:**

Keratoconus (KCN) is a disorder that usually appears during adolescence and progressively reduces visual acuity. KCN may lead to differences in personality features as a result of vision loss and the numerous clinical examinations and treatment methods used from a young age. The aim of this study was to better understand the psychological characteristics of KCN patients and to define possible correlations between corneal topographic parameters and psychological state.

**Methods:**

A total of 59 KCN cases were included in the study group and were compared with 65 age- and sex-matched healthy individuals. All of the participants underwent a routine ophthalmic examination that included corrected distance visual acuity (CDVA), biomicroscopy, and fundoscopy. The KCN patients were evaluated busing Scheimpflug corneal topography. Psychiatric evaluations were performed using the Eysenck Personality Questionnaire Revised-Short Form (EPQ), the Self-Confidence Scale, the Maudsley Obsessive-Compulsive Inventory (MOCI), and the Beck Depression Inventory (BDI).

**Results:**

The mean age of the case and control groups was 23.98±5.7 years and 25.82±5.4 years, respectively. The KCN cases had significantly higher EPQ neuroticism subscale scores; higher MOCI subscale scores, with the exception of the doubting subscale; and higher BDI scores. Analysis of the KCN duration revealed a positive correlation with the checking and slowness subscales of the MOCI, however, there was no significant correlation between the psychometric scale scores, corneal topographic parameters, and CDVA.

**Conclusion:**

A substantially asymmetrical course and a relatively long period for KCN to result in severe vision loss might explain the lack of correlations between psychological parameters and visual acuity. Nonetheless, the apparent effect of vision loss on emotional distress cannot be disregarded; the day-to-day progressive loss of visual acuity and multiple, costly interventions may initiate or contribute to a depressive mood in KCN patients. A vicious depressive cycle and the exhaustion of long-term coping mechanisms might be underlying factors for the higher neuroticism scores seen among KCN patients. Both the personality traits and mental state of KCN patients demonstrate distinguishing properties; clinicians working with these patients should consider their mental state in addition to other factors in order to achieve better treatment outcomes.

## Introduction

Keratoconus (KCN) is a disorder that usually starts during adolescence, progressively lowers visual acuity, and affects 70–80% of the human lifespan (1). It is still the most common cause of keratoplasty worldwide (2). There has been a major surge in incidence and prevalence rates of KCN, especially over the past 10 years (3). These patients are exposed to numerous clinical examinations and unusual treatment methods ever since their early ages.

The clinicians who deal with KCN patients emphasized that the management of these patients can be quite challenging (4). On the other hand, KCN patients believe that the attitude of health-care professionals toward them was not perfect either (5). This discordance validates the need for a better understanding of the psychological aspects of KCN patients. Even though typical KCN personality organization is yet to be defined; these patients have previously been reported as depressive, obsessive, and introverted (6, 7) Furthermore, case reports of psychotic symptoms among KCN patients give rise to the thought of a shared genetic base between psychiatric disorders and KCN (8, 9). It is also important to consider the differences in personality features of KCN patients which might take root from visual loss since the early ages.

In this study; personality features, predispositions toward several psychiatric disorders, and corneal topographic parameters of KCN patients were evaluated and compared to healthy controls of similar age group. Thus, we aimed to better understand the psychological characteristics of these patients and define the possible correlations between corneal topographic parameters and psychological states.

## Methods

### Study Sample

This cross-sectionally designed case–control study included a total of 124 participants, aged between 16 and 40, who were admitted to an ophthalmology outpatient unit of a tertiary university hospital, between July 1, 2020, and January 1, 2021. The G-Power analysis program (Faul, Erdfelder, Lang and Buchner, 2007; version 3.1) was used to calculate the sample size. Type I Error 0.05, Type II Error: 0.10, 1-β (power): 90% of the sample size was calculated as 55 for each group. Individuals who had additional ophthalmologic disorders such as cataracts, glaucoma, uveitis, and history of herpetic eye disease, deep corneal scar, untreated eyelid disorders, pregnancy, breastfeeding, and without the mental capacity to fully understand and execute the instructions of psychiatric scales were excluded from the study. A total of 59 patients who were diagnosed as “KCN compatible” by Scheimpflug corneal topography software which was based on keratometer and pachymetry measures were included in the KCN (case) group. The healthy control group comprised a total of 65 participants who applied to the ophthalmology clinic for a routine eye examination and had no previous history of any systemic or mental disorders. Individuals in the control group were matched by gender and age with KCN patients.

This research has been approved by the ethics committee of Recep Tayyip Erdogan University Faculty of Medicine (approval number: 40465587-050.01.04-147, Date: June 24, 2020) and researchers agreed to comply with the tenets of the Declaration of Helsinki. All participants and the parents of the participants who are under the age of 18 gave their informed consent before their inclusion in the study.

### Ophthalmic Examination

All participants underwent a routine ophthalmic examination including spectacle-corrected distance visual acuity (CDVA) with Snellen chart, tonometry, biomicroscopy, and fundoscopy. The KCN patients were examined by Scheimpflug corneal topography (Sirius, CSO, Italy). The mean and maximum keratometry (Kmean-Kmax), minimum central corneal thickness (MCCT), cylindric diopter (Cyl D) keratometry vertex front and back (KVf-KVb), corneal aberrations (total, high order, spherical, coma, and trefoil), CDVA, Amsler-Krumeich stage, and KCN duration values were recorded for statistical analysis.

### Psychiatric Evaluation

#### Eysenck Personality Questionnaire-Revised Short Form (EPQ-RSF)

It includes a total of 24 items covering three main personality features (sub-scales) such as “neuroticism,” “extraversion,” and “psychoticism.” Furthermore; a “lie” sub-scale was added to prevent bias during administration and control the validity. Each sub-scale was evaluated with 6 true (1)-false (0) questions. EPQ-RSF was found to be a valid and reliable scale in Turkish language (10).

#### Self-Confidence Scale (SCS)

Akin developed this scale in Turkish language to assess the self-confidence features of healthy individuals and psychiatric patients (11). It is a 5-point Likert-type self-report scale that evaluates two dimensions of self-confidence: Internal (which includes knowing and loving oneself, setting, and recognizing clear goals) and external (which includes social communication skills, rightfully expressing oneself, taking risks, and controlling emotions) self-confidence.

#### Maudsley Obsessional-Compulsive Inventory (MOCI)

MOCI is a self-report scale that was originally developed by Hodgson and Rachman to assess the type and extensity of obsessional and compulsive symptoms in healthy individuals and psychiatric patients (12). The original form included a total of 30 items (in the form of true-false questions) which explored the dimensions of “cleanliness,” “checking,” “slowness,” and “doubting.” Adaptation, validity, and reliability study of MOCI in the Turkish language was done by Erol and Savaşır; and additional 7 items which explored the dimension of “rumination” were included in the Turkish version. Participants might score between 0 and 37 and there was no determined cutoff score for the Turkish version of MOCI (13).

#### Beck Depression Inventory (BDI)

This self-report inventory consists of 21 items that quantitatively explore the self-perceived depressive symptoms in vegetative, emotional, cognitive, and motivational areas of depression. Higher scores indicate a more severe depressive mood (14). Validity and reliability study of this inventory among Turkish population was done by Hisli and it is a widely used scale in evaluation of the severity of depressive symptoms (15).

### Statistical Analysis

The statistical analysis was performed by SPSS 21.0 (IBM Corporation, 2018). The mean and standard deviation (±SD) values were given for continuous data; whereas numbers and percentages were given for categorical data. Kolmogorov–Smirnov test was used to check whether the continuous data were normally distributed. To compare continuous data between groups; independent t-test was used for parametric and Mann-Whitney U (MWU)-test was used for non-parametric data. Categorical data were analyzed using Chi-square or Fisher’s exact test. P<0.05 was accepted as statistically significant.

## Results

**Figure 1.**
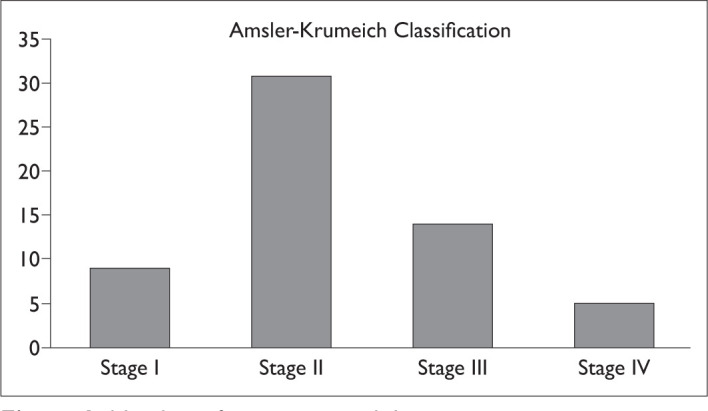
Number of patients in each keratoconus stage.

There was no statistically significant difference between groups regarding age (t=−1.840, p=0.068, independent t-test). However, corneal collagen cross-linking (CXL) performed (CXL+) patients were significantly younger than those who were not (CXL−). Regarding Amsler-Krumeich KCN stages of case group; 15.3% (n=9) was on “Stage 1,” 52.5% (n=31) was on “Stage 2,” 23.7% (n=14) was on “Stage 3,” and 8.5% (n=5) was on “Stage 4” (Fig. 1). The mean KCN duration of the cases was 25.64 (±19.63) months. Results of statistical analyses of age, gender between groups, and ophthalmological parameters of KCN cases are summarized in Tables 1 and 2.

**Table 1.** Comparisons of age, gender, and ophthalmological parameters between keratoconus and control groups

	**Mean±SD**		**T**	**p^a^**
	**Case**	**Control**		
Age (years)	23.98±5.7	25.82±5.4	-1.840	0.068
CXL (+) (n=39)	22.30±4.5	–		
CXL (-) (n=20)	27.53±6.5	–	3.577	0.001
Unilateral (n=23)	24.43±4.7	–		
Bilateral (n=36)	23.69±6.3	–	0.479	0.634
	**Number (%)**		**χ^2^**	**p^b^**
	**Case**	**Control**		
Gender				
Male	41 (69.4)	40 (61.5)	0.990	0.320
Female	18 (30.6)	25 (38.5)		

^a^Independent T-Test. ^b^Chi-square test. SD: Standard deviation; CXL: Corneal cross-linking.

**Table 2.** Mean corneal topography parameters of keratoconus patients

**Topography parameters**	**Mean±SD**
MCCT	450.80±40.8
Kmean	47.11±3.1
Kmax	56.52±5.5
Cly D	-3.35±2.0
KVf	35.44±19.3
KVb	80.69±34.6
TOA	2.86±1.4
HOA	1.73±0.9
LOA	2.15±1.4
Coma	1.41±0.8
Trefoil	0.59±0.4
SpherAb	-0.01±0.35

MCCT: Minimum central corneal thickness; Kmax: Maximum keratometry; Kmean: Mean keratometry; ClyD: Cylindric diopter; KVf: Keratometer vertex front; KVb: Keratometer vertex back; TOA: Total ocular aberrations; HOA: High order aberrations; LOA: Low order aberrations; SpherAb: Spheric aberrations; SD: Standard deviation.

Psychiatric scale score differences were separately analyzed between case/control, CXL+/CXL−, and unilateral/bilateral KCN groups (Table 3). In the comparison of case and control groups; the case group had significantly higher scores only on the “neuroticism” subscale of EPQ (Z=−3.220, p=0.001, MWU test) and they did not differ on any of the other subscales of EPQ or SCS. The case group also had significantly higher scores on all of the MOCI subscales except for the “doubting” subscale (Z=−2.208 and p=0.027 for “cleanliness;” Z=−2.410 and p=0.016 for “checking;” Z=−2.620 and p=0.009 for “slowness;” Z=−3.451 and p=0.001 for “rumination;” Z=−2.838 and p=0.005 for total MOCI scores, MWU tests). In addition, the case group scored significantly higher in BDI (Z=−3.093, p=0.002, MWU test) compared to the control group. There were no statistically significant differences on any of the scales when CXL (−) and CXL (+) groups were compared. Unilateral and bilateral KCN patients had statistically significant difference only on the “extraversion” subscale of EPQ (Z=−2.104, p=0.035, MWU test) and they did not differ on any other psychiatric scale scores (Table 3).

**Table 3.** Comparisons of psychiatric scale scores between groups

	**Mean Rank**		**Z**	**p^a^**	**Mean Rank**		**Z**	**p^a^**	**Mean Rank**		**Z**	**p^a^**
	**Case (n=59)**	**Control (n=65)**			**CXL-(n=19)**	**CXL+ (n=40)**			**Unlilat (n=23)**	**Bilat (n=36)**		
EPQ		
Neurotism	73.73	53.10	-3.220	**0.001**	34.32	27.95	-1.349	0.177	32.54	28.38	-0.922	0.357
Extraversion	60.62	65.20	-0.717	0.473	32.00	29.05	-0.627	0.530	24.22	33.69	-2.104	**0.035**
Psychotism	58.63	67.03	-1.333	0.183	34.84	27.70	-1.548	0.122	27.83	31.39	-0.806	0.420
Lie	58.36	67.28	-1.422	0.155	35.21	27.53	-1.644	0.100	34.17	27.33	-1.527	0.127
SCS												
Internal	60.82	65.02	-0.648	0.517	31.45	29.31	-0.446	0.655	29.07	30.60	-0.334	0.738
External	61.70	64.20	-0.386	0.700	31.76	29.16	-0.544	0.586	29.61	30.25	-0.140	0.889
Total	61.03	64.82	-0.583	0.560	31.34	29.36	-0.414	0.679	29.39	30.39	-0.218	0.828
MOCI												
Cleanliness	70.38	56.19	-2.208	**0.027**	35.66	27.31	-1.763	0.078	31.96	28.75	-0.707	0.480
Checking	71.03	55.58	-2.410	**0.016**	32.47	28.83	-0.771	0.441	30.70	29.56	-0.251	0.802
Slowness	71.56	55.10	-2.620	**0.009**	35.82	27.24	-1.839	0.066	28.59	30.90	-0.518	0.604
Doubting	67.02	59.29	-1.222	0.222	30.53	29.75	-0.166	0.868	27.07	31.88	-1.073	0.283
Rumination	74.52	52.37	-3.451	**0.001**	34.34	27.94	-1.353	0.176	28.61	30.89	-0.503	0.615
Total	72.56	54.18	-2.838	**0.005**	34.08	28.06	-1.259	0.208	29.63	30.24	-0.132	0.895
BDI	73.42	53.38	-3.093	**0.002**	35.82	27.24	-1.795	0.073	30.13	29.92	-0.047	0.963

^a^Mann-Whitney U Test, statistically significant p-values are written in bold. EPQ: Eysenck Personality Questionnaire; SCS: Self-Confidence Scale; MOCI: Maudsley Obsessional–Compulsive Inventory; BDI: Beck Depression Inventory; CXL-: Keratoconus who did not receive Cross-Linking; CXL+: Keratoconus who received Cross-Linking; Unilat: Unilateral keratoconus patients; Bilat: Bilateral keratoconus patients.

Correlations between psychiatric scale scores and ophthalmological parameters were also explored; and there was a low degree of positive correlation between Kmax and “psychoticism” subscale of EPQ (r=0.284, p=0.029), low degree of positive correlation between MCCT and “internal self-confidence” subscale of SCS (r=0.269, p=0.039), a moderate degree of negative correlation between spherical aberration (SpherAb) and “external self-confidence” subscale of SCS (r=−0.304, p=0.019), low degree of positive correlation between duration of KCN and “checking” subscale of MOCI (r=0.278, p=0.033), and a moderate degree of positive correlation between duration of KCN and “slowness” subscale of MOCI (r=0.334, p=0.010). Results of correlation analyses are detailed in Table 4. In addition, we could not determine any correlation between the KCN stage and psychiatric scale scores (Table 4).

**Table 4.** Correlations between ophthalmological parameters and psychiatric scales of cases

**Correlationsa**		**Eysenck Personality Questionnaire**				**Self-Confidence Scale**			**Maudsley Obsessional Compulsive Inventory**						**BDI**
		**E**	**L**	**N**	**P**	**SCS-I**	**SCS-E**	**Total**	**Che**	**Cle**	**Slow**	**Doub**	**Rum**	**Total**	
CDVA	r	-.010	.029	.073	-.176	.164	.142	.164	.049	.075	.060	.047	-.005	.075	-.237
	p	.939	.826	.585	.181	.216	.283	.214	.712	.570	.649	.724	.968	.571	.071
Duration of KCN	r	-.147	-.113	.178	-.138	-.131	-.148	-.149	.278	.022	.334	.236	.200	.189	.187
	p	.265	.393	.176	.298	.324	.264	.259	.033	.869	.010	.072	.128	.152	.156
Stage	ρ	.038	.011	-.112	.075	-.021	-.039	-.056	-.094	-.139	-.054	-.108	-.105	-.164	-.056
	p	.773	.933	.397	.574	.873	.767	.675	.477	.295	.685	.416	.429	.215	.674
MCCT	r	-.075	.036	-.023	-.240	.269	.132	.216	-.056	.038	-.155	-.115	-.096	-.053	-.129
	p	.572	.788	.861	.068	.039	.320	.101	.675	.776	.242	.386	.471	.692	.332
Kmax	r	.065	-.084	.020	.145	.020	.075	.050	.113	.037	-.007	.064	.025	.040	.072
	p	.623	.528	.880	.274	.883	.573	.704	.394	.778	.957	.632	.849	.766	.590
Kmean	r	-.059	-.120	-.077	.284	.047	-.013	.018	-.002	.104	.011	.058	-.048	.034	.028
	p	.656	.365	.562	.029	.725	.921	.891	.987	.435	.936	.663	.718	.801	.831
ClyD	r	-.128	.163	.042	.076	-.046	-.088	-.072	-.025	-.161	-.068	-.092	-.071	-.092	-.082
	p	.334	.219	.750	.566	.727	.509	.588	.848	.223	.608	.486	.593	.490	.537
KVf	r	.109	-.045	-.099	.055	.088	.060	.080	.100	-.017	.080	.099	.070	.055	.037
	p	.413	.735	.456	.682	.509	.649	.549	.453	.900	.545	.456	.599	.680	.783
KVb	r	.027	.027	-.115	.057	.088	.033	.065	.096	-.066	.037	.003	.061	.007	.047
	p	.837	.838	.386	.667	.510	.804	.625	.471	.617	.782	.982	.646	.959	.722
TOA	r	.157	-.089	-.062	.068	.076	.124	.108	.072	.129	.071	.107	.047	.087	.067
	p	.235	.501	.643	.610	.565	.349	.418	.589	.331	.595	.420	.724	.512	.616
HOA	r	.161	-.085	-.067	.045	.045	.074	.064	.063	.049	-.004	.123	-.046	.029	.011
	p	.225	.523	.612	.737	.735	.577	.631	.638	.713	.977	.354	.732	.826	.933
LOA	r	.141	-.087	-.026	.077	.080	.134	.114	.074	.150	.111	.094	.104	.113	.096
	p	.287	.513	.843	.562	.549	.312	.389	.0577	.257	.401	.480	.434	.396	.471
Coma	r	.189	-.091	-.109	-.001	.119	.151	.145	.037	.028	.028	.106	-.089	.009	-.072
	p	.152	.491	.410	.994	.370	.254	.274	.783	.836	.836	.425	.501	.949	.586
Trefoil	r	.152	-.078	.090	.062	.-115	.032	-.045	.085	.043	-.075	.129	-.026	.013	.163
	p	.250	.558	.496	.641	.385	.808	.735	.522	.747	.572	.331	.844	.919	.218
SpherAb	r	-.229	-.013	-.047	-.002	-.130	-.304	-.233	.029	.119	.061	-.030	-.007	.064	.077
	p	.081	.921	.722	.987	.327	.019	.076	.825	.368	.648	.821	.959	.630	.564

^a^Pearson Correlation Test was used for continuous data and Spearman Correlation Test was used for ordinal data; statistically significant p-values are written in bold. E: Extraversion; L: Lie; N: Neurotism; P: Psychotism; SCS-I: Internal Self-confidence; SCS-E: External Self-confidence; Che: Checking; Cle: Cleanliness; Slow: Slowness; Doub: Doubting; Rum: Rumination; CDVA: Corrected Distance Visual Acuity; KCN: Keratoconus; r: Pearson Correlation Coefficient; ρ: Spearman Correlation Coefficient; MCCT: Minimum central corneal thickness; Kmax: Maximum keratometry; Kmean: Mean keratometry; ClyD: Cylindric diopter; KVf: Keratometer vertex front; KVb: Keratometer vertex back; TOA: Total ocular aberrations; HOA: High order aberrations; LOA: Low order aberrations; SpherAb: Spheric aberrations.

## Discussion

In our study, we determined significantly higher depressive and obsessional-compulsive symptom scores among patients with KCN. However, we could not observe any difference between the case and control groups regarding their SCS scores. As for their personality features, “neuroticism” scores were significantly higher in KCN patients. There was a positive correlation between KCN duration and two obsessional symptom scores (checking and cleanliness). Furthermore, there were correlations between psychiatric scale scores and Kmean, SpherAb, and MCCT parameters; whereas visual acuity and KCN stage were not correlated to any of the psychological scales.

Even though the exact mechanism is widely unknown, the “two-hit” hypothesis was proposed to explain the development of KCN (16). According to this hypothesis, “first hit” is related to genetic disposition forming a basis for the development of KCN. “The second hit” includes environmental factors which are related to the progression and severity of the disease. Similarly, Mannis et al. proposed a “two-hit” hypothesis for KCN personality: Visual loss forms the basis of “first hit;” whereas disparate perceptions toward the course of KCN which these patients face in the provider’s office make way for a “second hit” (17). Besides, the individual’s disabilities in daily life activities due to diminished visual acuity also cause elevated emotional stress which might result in cognitive dysfunction and psychosis. Interestingly, “quality of life” scores of KCN patients were found lower independent of their levels of visual acuity (18). The KCN has a substantially asymmetrical course and relatively long periods are needed to progressively afflict both eyes and result in severe visual loss; so this might explain the lack of correlations between psychological parameters and visual acuity that we observed in our study.

The previous studies have stated that it is considerably hard to define a specific personality feature for KCN patients (17). Apart from its onset during adolescence, various other factors related to KCN might have an impact on personality features. However, several studies reported particular psychological problems among KCN patients (17, 19, 20). In line with these, we also found that KCN patients had significantly higher depressive symptom and “neuroticism” scores; but neither of them had a relationship with any of the topographic parameters or visual acuity. Similarly, Moschos et al. also reported that depression among KCN patients was more frequent and severe but it was not related to the level of visual acuity (7). Furthermore, depressive symptom scores were reported to be higher in various other eye diseases such as retinitis pigmentosa, age-related macular degeneration, glaucoma, and Stargardt disease (21-24). Even though the apparent effect of visual loss on emotional distress cannot be discarded; day-to-day progressive loss of visual abilities and costly interventions which have limited success rates might set off a depressive mood in KCN patients. Depressive vicious cycle and weariness caused by long-term coping mechanisms might be the underlying factors for neurotic personality features seen among KCN patients. Limited case series have proposed shared genetic mechanisms for schizophrenia and KCN comorbidity; however, there are no data on the shared genetic mechanisms between depression and KCN (8). Further studies in this aspect might shed light on possible common genetic factors between these two conditions.

Although Cingu et al. stated that CXL treatment caused significant improvement in anxiety symptoms; we could not determine any difference between patients who received CXL and those who did not regarding their psychological parameters (25). This might be due to the methodology of our study in which we cross-sectionally examined these profiles and did not compare pre-CXL and post-CXL scores. Moreover, in this research, the mean age of patients who underwent CXL treatment was significantly lower than patients who did not. The CXL treatment became popular over the past 10 years and worsening of KCN symptoms is relatively less common in elderly patients, so these factors might account for the age disparity between the two groups. Interestingly, we observed that “extraversion” scores of bilateral KCN patients were significantly higher compared to unilateral KCN patients. Besides, even though they were not statistically significant, we found a trend toward higher SCS and lower depressive symptom scores among bilateral KCN patients. Having bilateral KCN might push these patients to seek treatment and help more frequently compared to unilateral KCN patients. In addition, bilateral KCN patients may have higher needs for compensating for their more severe visual disabilities; so they may be obliged to benefit from social support systems (i.e., other people in their community) more often, all of which constitute for a more “extrovert” personality structure.

Nowadays, extensive use of anterior segment optic coherence tomography and Scheimpflug camera-based advanced corneal topography devices has enabled early diagnosis of KCN (26). Furthermore, it is possible to accomplish favorable results in visual skills using new generation hybrid and scleral contact lenses and non-penetrating keratoplasty methods (27). In our study, we found significant positive correlations between duration of KCN and “checking” and “slowness” subscales of MOCI; which might be explained by administration of relatively up-to-date treatment methods (such as CXL) among patients who are younger and at early stages of the KCN. However, the majority of our KCN patients were at relatively early stages (only 8% of our participants were at Stage IV) and this might be the reason for the lack of difference regarding MOCI scores between CXL (+) and CXL (−) groups. In addition, all of the MOCI scores except for “doubting” were significantly higher in our case group. Eye rubbing is accepted as a major risk factor for KCN and proposed to be related to the externalization of emotional stress during adolescence (6, 28). Eye surface irregularity caused by corneal apical protrusion results in eye surface inflammation which is also exacerbated by blinking and eye rubbing (29). These conditions might contribute to a more “obsessive” personality structure; however, we could not determine any correlation between MOCI scores and topographic parameters.

Despite its strength as being one of the limited studies in this field, our research has some limitations as well. First, the majority of our KCN patients were in Stages I and II (relatively earlier stages) and comparisons between more equally distributed groups might give better results for psychological features according to stages of this condition. Second, evaluation of attitudes of clinicians toward KCN patients might shed light on the effects of the relationship between patients and physicians on the psychological well-being and personality features. Third, we did not use any semi-structured psychiatric interviews, so the observations in our study reflect only the symptomatology rather than the exact psychiatric diagnoses. Besides, administration of “quality of life” scales may be useful to explain the causality between topographic and psychological parameters. However, it should be kept in mind that, due to their relatively depressive moods, these patients may have a hard time complying with time consuming questionnaires. Moreover, consecutive and overwhelming administrations of different questionnaires may result in inconsistent findings.

## Conclusion

KCN patients become acquainted with this condition at an early age and face the risk of progressive visual loss. The necessity for long years of regular clinical follow-ups and costly treatment modalities might make them vulnerable to psychological adversities. We found that KCN patients had significantly higher “neuroticism,” depressive and obsessional symptom scores; but none of them was correlated with visual acuity and topographical parameters. However, it was shown that “checking” and “slowness” obsessional symptom scores increased as the duration of KCN got longer. By keeping the mental and psychological vulnerabilities in mind, clinicians who deal with this sensitive group might achieve better treatment outcomes and patient satisfaction.

### Disclosures

**Ethics Committee Approval:** Recep Tayyip Erdogan University Faculty of Medicine Ethics Committee, protocol number: 40465587-050.01.04-147, Date: 24.06.2020.

**Peer-review:** Externally peer-reviewed.

**Conflict of Interest:** None declared.

**Authorship Contributions:** Involved in design and conduct of the study (MGA, MB, ÇH), preparation and review of the study (MGA, MB, HA, MFS, ÇH), data collection (MGA, MB, HA, MFS) and statistical analysis (MGA, MB).
